# THE ASSOCIATION OF HEARING LOSS AND FRAILTY AMONG OLDER ADULTS IN THE US

**DOI:** 10.1093/geroni/igad104.0544

**Published:** 2023-12-21

**Authors:** Pablo Martinez Amezcua, Sahar Assi, Wuyang Zhang, Nicholas Reed

**Affiliations:** Johns Hopkins Bloomberg School of Public Health, Baltimore, Maryland, United States; Johns Hopkins Bloomberg School of Public Health, Epidemiology, Baltimore, Maryland, United States; Johns Hopkins Bloomberg School of Public Health, Epidemiology, Baltimore, Maryland, United States; Johns Hopkins Bloomberg School of Public Health, Baltimore, Maryland, United States

## Abstract

Frailty is common in older adults and increases the risk of functional limitations and death. Hearing loss affects ~2/3 of older adults in the U.S. and is associated with poorer physical and cognitive function and social isolation, which may increase the risk for frailty. We aimed to determine if hearing loss is associated with higher odds of being frail or pre-frail versus robust and if hearing aid use is protective. We analyzed data from 2,361 participants from the National Health and Aging Trends Study (NHTAS), a U.S. nationally representative sample of older adults, collected in 2021. Hearing was assessed by pure-tone audiometric testing, and we used a speech-frequency (0.5-4 kHz) better hearing ear’s pure-tone average (BPTA) to categorize participants into no hearing loss (BPTA≤25 dB HL), mild (26-40), and moderate or greater (>40) hearing loss. Physical frailty was categorized according to the presence of the frailty phenotype components (unintentional weight loss, exhaustion, low physical activity, weakness, slow walking speed) into robust (0), pre-frail (1-2), and frail (≥3). In our study population (mean age=81, 56% female, 19% Black), there were 1,724 (73%) participants with hearing loss. Using multinomial logistic regression, we found that participants with moderate or greater hearing loss (vs. those without) had higher odds of being frail versus robust (OR=1.82, 95% CI 1.11, 2.99, p-trend=0.016). Hearing loss may be a risk factor for frailty. Future studies on the impact of hearing interventions in preventing and managing frailty will have significant public health and clinical importance.

